# Dilated convolution capsule network for apple leaf disease identification

**DOI:** 10.3389/fpls.2022.1002312

**Published:** 2022-11-01

**Authors:** Cong Xu, Xuqi Wang, Shanwen Zhang

**Affiliations:** School of Electronic Information, Xijing University, Xi’an, China

**Keywords:** apple leaf disease identification, dilated convolution, capsule network (CapsNet), dilated convolution CapsNet (DCCapsNet), inception

## Abstract

Accurate and rapid identification of apple leaf diseases is the basis for preventing and treating apple diseases. However, it is challenging to identify apple leaf diseases due to their various symptoms, different colors, irregular shapes, uneven sizes, and complex backgrounds. To reduce computational cost and improve training results, a dilated convolution capsule network (DCCapsNet) is constructed for apple leaf disease identification based on a capsule network (CapsNet) and two dilated Inception modules with different dilation rates. The network can obtain multi-scale deep-level features to improve the classification capability of the model. The dynamic routing algorithm is used between the front and back layers of CapsNet to make the model converge quickly. In DCCapsNet, dilated Inception instead of traditional convolution is used to increase the convolution receptive fields and extract multi-scale features from disease leaf images, and CapsNet is used to capture the classification features of changeable disease leaves and overcome the overfitting problem in the training network. Extensive experiment results on the apple disease leaf image dataset demonstrate that the proposed method can effectively identify apple diseases. The method can realize the rapid and accurate identification of apple leaf disease.

## Introduction

Apple is one of the most popular fruits. However, it is often affected by various diseases, which reduce its yield and quality (Pandiyan et al., 2020). Rapid and accurate detection and identification of these diseases is a prerequisite for disease control and accurate use of pesticides. Traditional methods of manual detection and identification of apple diseases mainly rely on visual recognition, which is not only subjective but also time-consuming, laborious, and inefficient and requires sufficient field experience and subjective assumptions. This method cannot be used for the quantitative identification of diseases; nor can it be widely used in large apple plantations. Apple leaves are susceptible to diseases. Because of the complex symptoms of apple leaf disease, detection and identification by apple disease leaf image is challenging research (Mishra et al., 2017; Puspha Annabel et al., 2019). Zhang et al. (2017) proposed an apple leaf disease recognition method based on image processing techniques and pattern recognition, including image lesion segmentation, feature extraction, dimension reduction, and disease identification. In the method, 38 classifying features of color, texture, and shape were from each segmented spot image, and the few most valuable features were selected by combining genetic algorithm (GA) and correlation feature selection algorithm. Finally, the diseases were recognized by a support vector machine (SVM) classifier. In fact, the similarity between the different-class disease spot images is small, while the similarity between the within-class disease spot images is largely due to the complex background environment, so the traditional apple leaf disease recognition using complex image pretreatment and feature extraction cannot guarantee a high disease recognition rate.

With the development of deep learning and big data processing technologies, convolutional neural networks (CNNs) realize end-to-end detection by learning multi-level features of different receptive fields, scenes, and scales (Lei et al., 2018; Li et al., 2019; Sun et al., 2021) and have become a topic of research in the crop automatic disease recognition fields (Sun et al., 2017). Sun et al. (2021) proposed a lightweight CNN model to detect apple leaf diseases in real time. They constructed a dataset of apple leaf disease image dataset, namely, AppleDisease 5, proposed a MEAN block, and built an apple leaf disease detection model by using the MEAN block and Apple-Inception module. Agarwal et al. (2019) developed a CNN model to identify apple disease. It consists of three convolution layers and three max-pooling layers followed by two densely connected layers. They tested the model with varying numbers of convolution layers from two to six and found that three layers have the best. Jiang et al. (2019) proposed an apple leaf disease real-time detection based on improved CNN. In the method, the apple leaf disease dataset was constructed *via* data augmentation and image annotation technologies, and an apple leaf disease detection method based on deep CNN (DCNN) was proposed by introducing the GoogLeNet Inception structure and Rainbow concatenation. The proposed model was trained using a dataset of 26,377 images of diseased apple leaves to detect these five common apple leaf diseases. Yan et al. (2020) proposed an improved VGG16 model, namely, VGG-ICNN, for apple leaf disease recognition. It consists of approximately 6 million parameters that are substantially fewer than most of the available high-performing deep learning models. Zhong et al. (Zhong and Zhao, 2020) proposed DenseNet-121 to identify apple leaf diseases and used an apple leaf image dataset including 2,462 images of six apple leaf diseases to train and evaluate the model. Some deep learning approaches have recently been introduced for leaf disease identification, such as VGG and residual network (ResNet). Son et al. (Yu and Son, 2020) proposed a deep learning architecture for apple disease recognition by considering the leaf spot attention mechanism. To realize this, they designed a feature segmentation subnetwork to provide more discriminative features and a spot-aware classification subnetwork for the feature segmentation subnet and then trained through early fusion and late fusion to generate semantic point feature information. The results proved that the proposed method outperforms conventional state-of-the-art deep learning models. Luo et al. (2021) proposed an apple disease classification model based on a multi-scale conventional ResNet. To solve the problem of serious loss of information in the ResNet downsample, the channel projection and spatial projection of downsample were separated, the 3 × 3 convention in ResBlocks was replaced by pyramid convolution, and the dilated convolution with different dilation rates was introduced into pyramid convolution to enhance the output scale of feature maps and improve the robustness of the model. The results on the dataset of this paper demonstrated that the optimal model has a high accuracy, which can provide a reference for the prevention and control of apple leaf diseases. Khana et al. (2022) proposed a real-time apple leaf disease detection system based on deep learning. The qualitative results validated that the proposed system can efficiently and accurately identify leaf disease symptoms and can be used as a practical tool by farmers and apple growers to aid them in the diagnosis, quantification, and follow-up of infections. Di et al. (Di and Li, 2022) proposed an apple disease detection approach based on improved CNN, namely, DF-Tiny-YOLO. Feature reuse is combined with DenseNet dense connection network to reduce the disappearance of depth gradient, so as to strengthen feature propagation and improve detection accuracy. The calculation parameters of DF-Tiny-YOLO are reduced by convolution kernel compression, and the operation detection speed is improved. Feature fusion is realized by feature superposition. The results showed that this method can improve detection performance significantly.

According to the above methods, the deeper the convolution layer is, the more abstract the extracted features are, and the higher the recognition rate is. However, the larger convolution kernel and the deeper CNN model have more training parameters, requiring longer training time and greater computational power.

Most of the existing apple detection models based on CNN are difficult to use on hardware resource platforms with limited computing capacity and storage capacity due to too many parameters. To improve the performance and adaptability of the existing apple detection model under the condition of limited hardware resources, while maintaining detection accuracy, reducing the calculation of the model and the model computing and storage footprint, and shortening detection time, Xia et al. (2020) proposed an apple detection model based on lightweight anchor-free deep CNN, namely, lightweight MobileNetV3. MobileNetV3 outperforms CenterNet and SSD (Single Shot Multibox Detector) in comprehensive performance, detection accuracy, capacity, and convergence speed. Li et al. (2022) proposed an apple identification method based on lightweight RegNet. To evaluate the effectiveness of this method, a series of comparative experiments were conducted using 2,141 images of five field apple leaf diseases and compared with the state-of-the-art improved CNN such as ShuffleNet, EfficientNet-B0, MobileNetV3, and Vision Transformer. The results show that the performance of RegNet-Adam is better than that of other pre-training models, and transfer learning can realize fast and accurate identification of apple leaf diseases.

In CNN, pooling is usually used to increase the receptive field and reduce the amount of calculation, but some useful information may be lost. Dilated convolution can increase the receptive field of the convolution kernel without increasing the number of parameters to improve the feature resolution, and the size of the output feature map can remain unchanged (Ahmed, 2021). Dilated convolution can be used to improve the quality of the training results and decrease the required computational costs. For example, a 3 × 3 convolution kernel with an expansion rate of 2 has the same receptive field as a 5 × 5 convolution kernel, while the number of parameters is only 9, which is 36% of the number of 5 × 5 convolution parameters. Therefore, dilated convolution can be used for constructing a lightweight CNN model (Fang et al., 2019). Thakur et al. (2022) introduced a lightweight CNN, namely, VGG-ICNN, for the identification of crop diseases using plant-leaf images. It consists of approximately 6 million parameters that are substantially fewer than most of the available high-performing deep learning models. Many models with large parameters have difficulty providing an accurate and fast diagnosis of apple leaf pests and diseases on mobile terminals. Zhu et al. (2022) proposed a lightweight model for early apple leaf pests and disease classification, where a LAD-Inception is built to enhance the ability to extract multi-scale features of different sizes of disease spots. Li et al. (2022) proposed a lightweight convolutional neural network RegNet to realize the rapid and accurate identification of apple leaf disease and conducted a series of comparative experiments based on 2,141 images of five apple leaf diseases (rust, scab, ring rot, panonychus ulmi, and healthy leaves) in the field environment.

CNN has a strong feature extraction ability, but it cannot acquire the relationship between feature attributes, such as relative position and size. Its high recognition rate on the complex image dataset depends on a large number of training samples, but the actual amount of data obtained is often limited, leading to the overfitting of CNN. Capsule Network (CapsNet) can make up for the deficiency of CNN. Capsule is a set of neurons that capture various parameters of a particular feature, each representing various properties of a particular entity that appears in an image. These attributes include many different types of instantiation parameters such as posture (position, size, and direction), deformation, speed, hue, and texture. One special property in the capsule is the presence of an instance of a category in the image. CapsNet transforms the scalar output of neurons into vector output, which is the probability of the entity’s existence. It not only can represent whether the image has a certain feature but also can represent the physical features such as rotation and position of the feature (Wang et al., 2019). Xiang et al. (2018) designed a multi-scale CapsNet (MS-CapsNet), in which the multi-scale features are extracted by multi-scale convolutional kernels and then used to construct the multi-dimensional primary capsules. Deng et al. (2018) used the improved double-layer CapsNet to classify the PaviaU (PU) dataset of hyperspectral images and obtained a recognition rate of 93.45%. Yang et al. (2018) compared the classical CNN with CapsNet in terms of network structure, parameter update, and training results. Experimental results showed that CapsNet is better on gray images than the classical CNNs. CNN-based architectures have performed amazingly well for disease detection in plants but at the same time lack rotational or spatial invariance. CapsNet addresses these limitations of CNN architectures. Janakiramaiah et al. (2021) proposed a variant of CapsNet called Multilevel CapsNet to characterize the mango leaves tainted by anthracnose and powdery mildew diseases. It is validated on a dataset of mango leaves collected in the natural environment.

Inspired by dilated convolution, MS-CapsNet, and their improvement, a dilated convolution capsule network (DCCapsNet) is constructed for apple leaf disease identification. The main contributions are given as follows:

- Two dilated Inception modules are introduced into CapsNet to extract the multi-scale classifying features of disease leaf images, improve the classification capability of the model, and overcome the overfitting problem.
- DCCapsNet is constructed to recognize apple leaf diseases, where the dynamic routing algorithm is used between the front and back layers of CapsNet to make the model converge quickly.
- The effectiveness of this method is verified by many experiments.

The rest of this paper is organized as follows. Section 2 briefly introduces dilated convolution and CapsNet. DCCapsNet is introduced in detail in Section 3. The experiments and analysis are presented in Section 4. The summary and prospect of the paper are given in Section 5.

## Related works

In this section, dilated convolution and CapsNet are briefly introduced.

### Dilated convolution

**Figure 1:**
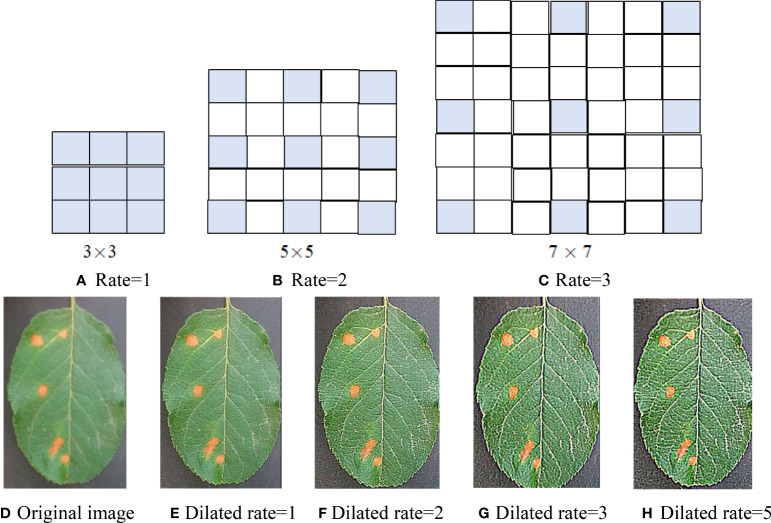
Dilated convolution with three dilation rates. **(A)** Rate = 1. **(B)** Rate = 2. **(C)** Rate = 3. **(D)** Original image. **(E)** Dilated rate = 1. **(F)** Dilated rate = 2. **(G)** Dilated rate = 3. **(H)** Dilated rate = 5.

Dilated convolution can enlarge the receptive field of the convolution layer by filling 0 in the middle of the convolution kernel, without increasing network parameters and then avoiding feature loss caused by pooling operation in CNN. Dilated convolution structures of three dilated rates are shown in **Figures 1A–C**, where (A) the receptive field is 3 × 3 with an expansion rate of 1 (that is, the traditional convolution kernel of 3 × 3); (B) the receptive field is enlarged to 5 × 5 with a dilated rate of 2 by filling with a 0 in the 3 × 3 standard convolution; (C) the receptive field is increased to 7 × 7 with a dilated rate of 3 by filling with two 0 in the 3 × 3 standard convolution. As can be seen from **Figures 1A–C**, with the increase of dilated rate, the size of the receptive field increases, but the network parameters do not increase, that is, nine parameters. Therefore, using the dilated convolutional instead of the traditional convolutional can extract more features without increasing the amount of computation.

Assume an apple rust leaf image and a 3 × 3 sharp kernel [−1 −1 −1;−1 9 −1;−1 −1 −1] and conduct several convolutions of the leaf image and dilated convolution kernels (*r* = 1, 2, 3, 5). The convolution maps are shown in **Figures 1E–H**. From the convolution maps in **Figure 1**, it can be seen that dilated convolution not only can expand the receptive field but also can extract more discriminant features than classical convolution and keep the relative spatial position of spot pixels unchanged without increasing computation and losing resolution. Comparing **Figures 1G, H**, there is not much difference between the two maps. Therefore, we utilized dilated convolution kernels (*r* = 1, 2, 3).

In DCNN, downsampling is usually used to increase the receptive field, but the image resolution will be reduced, resulting in the loss of spatial detail of the image. The dilated convolution expands the receptive field by setting the dilated rate, and setting different dilated rates can also capture multi-scale context information. It can be seen from **Figure 1**, on the basis of no additional parameters, that the receptive field of 3 × 3 convolution is expanded to 5 × 5 and 7 × 7, which can capture multi-scale features of the image. Therefore, multi-scale receptive fields can be obtained through the dilated convolution of different expansion rates. Dilated convolution can be considered a multi-scale convolution network. Dilated convolutional kernel and receptive field are calculated as follows


(1) $$\begin{array}{l} n=k+(k-1)(r-1)\\ l_{m}=l_{m-1}+[(f_{m}-1)\prod_{i=1}^{m-1}S_{i}] \end{array}$$

where *k* and *n* are the size of the original convolution kernel and dilated convolution kernel, respectively; *l*_*m*−1_ is the receptive field size of the (*m* − 1) layer; *l*_*m*_ is the receptive field size at the *m*th layer after the convolution of the void; *f*_*m*_ is the size of the convolution kernel at the *m*th layer; *S*_*i*_ is the step size of layer *l*.

### Capsule network

**Figure 2:**
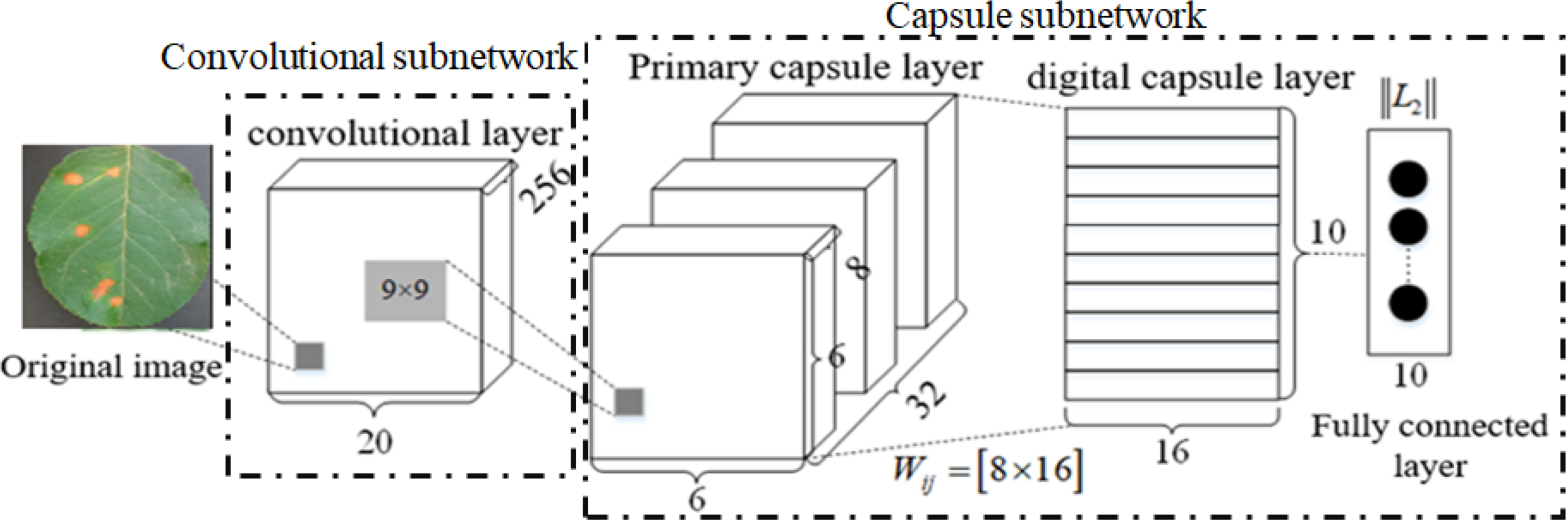
Architecture of capsule network (CapsNet).

CapsNet consists of one convolution layer and a primary capsule layer and a digital capsule layer. In its internal structure, the capsule layer is taken as the data processing unit, and the dynamic routing algorithm is adopted to transmit data between capsule layers, which has better feature expression ability than CNN. Its basic architecture is shown in **Figure 2**, where the convolution layer extracts the classifying features from the original images, the primary capsule layer mainly transforms the upper scalar representation to a vector representation and outputs a vector, and the digital capsule uses a dynamic routing algorithm to update the network parameters and avoids the loss caused by pooling. The final output is the eigenvector whose length is the probability that the test sample belongs to a certain class.

In **Figure 2**, *W* represents the weight. In a fully connected neural network, every neuron is a scalar (that is, there is only one numeric value), so every weight is just a scalar and a numeric value. However, in CapsNet, each capsule neuron is a vector (that is, it contains multiple values, such as [*x*_1_, *x*_2_, *x*_3_, …, *x_n_*]; the specific number *n* is designed according to the network), so the weight of each capsule neuron *W* should also be a vector. It is still updated according to backpropagation.

The input *s* of CapsNet is obtained as follows:


(2) $$s_{j}=\sum_{i}c_{ij}{\overset{\wedge }{u}}_{j|i},.{\overset{\wedge }{u}}_{j|i}=W_{ij}u_{i}$$

where *u* is the output of CapsNet of the upper layer and *W*_*ij*_ is the learnable weight matrix between the *i*th capsule and *j*th capsule; to be multiplied by each output, the coupling coefficient *c* added to the linear sum stage, is calculated by


(3) $$c_{ij}=\text{Soft​ max}(b_{ij})=\frac{\exp(b_{ij})}{\sum_{k}\exp(b_{ik})}$$

In the process of calculating *s* by forward propagation, *W* is set as a random value, *b* is initialized to 0, *u* is the output of the previous layer, and *s* of the next layer can be obtained. Sigmoid is often used as an activation function in FCN, while Squashing is an activation function. Its output *v* is as follows:


(4) $$v_{j}=\frac{\left||s_{j}^{2}|\right|}{1+\left||s_{j}^{2}|\right|}\frac{s_{j}}{\left||s_{j}|\right|}$$

In Sq. (4), the former part ||*s*_*j*_||^2^/(1+||*s*_*j*_||^2^) of the activation function is the scale of the input vector *s*, and the latter part *s*_*j*_/||*s*_*j*_|| is the unit vector of *s*. This activation function not only preserves the direction of the input vector but also compresses the modulus of the input vector to between [0, 1]. It is regarded as the probability of an entity’s appearance.

Dynamic routing is employed to update *b* and then update *c*, as follows:


(5) $$b_{ij}\leftarrow b_{ij}+{\overset{\wedge }{u}}_{j|i}\cdot v_{j}$$

Other convolution parameters of the entire network and *W* need to be updated according to the loss function, as follows:


(6) $$L_{c}=\;\sum_{k\in CNum}T_{k}\max(0,m^{+}-{\left||V_{k}|\right|}^{2})+\lambda (1-T_{k})max{\left(0,\left||V_{k}|\right|-m^{-}\right)}^{2}$$

where *m* ^+^ and *m* ^−^ are the category prediction values, *Λ* is the balance coefficient, *T*_*k*_ is the label of category, *T*_*k*_ = 1 is the correct label, *CNum* is the number of disease categories, *k* is the category number, and ||*V*_*k*_|| is the length of the vector representing the probability of discriminating as the class *k*th disease; the total loss is the sum of all digital capsule loss functions. The default values are set as *m* ^+^ = 0.9, *m* ^−^ = 0.1, and *Λ* = 0.5.

## Dilated convolution capsule network

**Figure 3:**
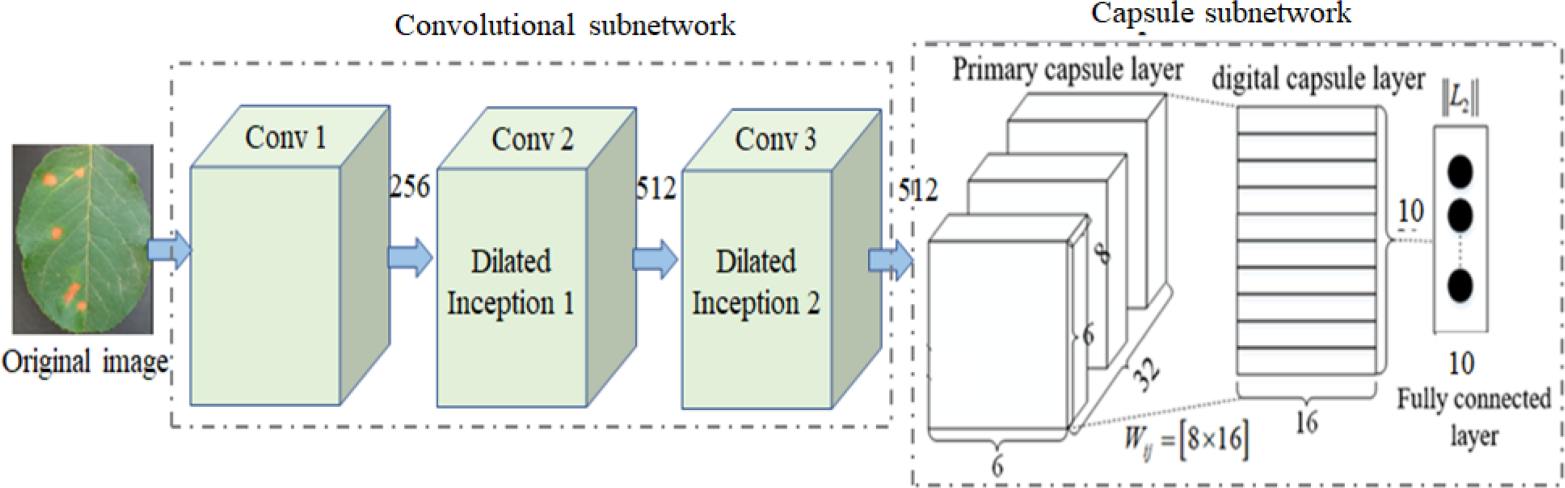
dilated convolution capsule network (DCCapsNet) architecture.

In complex image classification methods based on CNN and its variants, a large number of labeled training samples are usually required to train their parameters and improve their performance. However, it is very time-consuming to label a large number of samples. Although increasing network depth can improve the recognition rate, it means increasing network training time to optimize a large number of parameters. Traditional CapsNet only uses one convolution layer to extract the classification features, which cannot extract the deep multi-scale features from the complex images of disease leaves, resulting in low disease identification accuracy. To overcome the above problem, a DCCapsNet is constructed for apple disease recognition. Its architecture is shown in **Figure 3**, consisting of a convolution subnetwork and capsule subnetwork.

In DCCapsNet, Conv 1 of the convolution subnetwork is the same as the convolutional layer in CapsNet, and the capsule subnetwork is the same as the capsule layer in CapsNet, while Conv 2 and Conv 3 are two additional dilated Inception modules, which are introduced to enhance deep multi-scale feature extraction capability, thus improving the feature learning ability on complex disease leaf image dataset.

**Figure 4:**
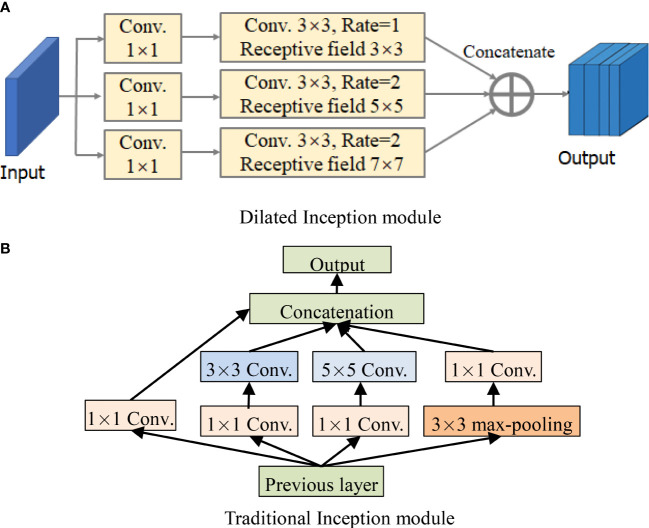
Structures of Inception module and dilated Inception module. **(A)** Dilated Inception module. **(B)** Traditional Inception module.

For the perception of the convolution kernel, the larger the convolution is, the stronger the ability of extracting disease information is. In fact, the lesions are smaller than the whole image, and other information on the image can be regarded as “noise”, which needs to be filtered. As a consequence, the dilated Inception module is designed as shown in **Figure 4A** (Janakiramaiah et al., 2021). The traditional Inception module is also shown in **Figure 4B** for comparison.

By comparing **Figures 4A, B**, it can be seen that DCCapsNet has more different receptive fields, such as 1 × 1, 3 × 3, 5 × 5, and 7 × 7. Since the 5 × 5 convolutions in **Figure 4B** are replaced by a 3 × 3 dilated convolution, the number of its convolution kernel parameters is smaller. The superiority of DCCapsNet is described as follows.

1. Adding two convolutional layers. The disease leaf images are often complex with irregular and multi-scale spots and contain an amount of healthy region and noise. To reduce the interference of useless information, the relationship between various features in the image can be fully connected, and the healthy region and noises can be filtered before entering the primary capsule layer. After Conv 1, Conv 2 and Conv 3 are added to reduce the interference caused by redundant information in complex backgrounds.
2. Dimension extension of capsules. After three convolutional modules, a large number of deep-level multi-scale features of the input images are extracted, and the extracted features are processed by the primary capsule layer and digital capsule layer and then compressed into capsules. The typical structure of the network is the capsule structure, which is the unit of storing information. When the dimension of the capsule structure is larger, there are enough storage units to store effective information in the network. Therefore, the network extends its dimension to 10D.
3. Intermediate capsule. In the capsule layer, the feature capsule at the bottom predicts the feature of the upper layer by attitude relation and then activates the upper layer by dynamic routing algorithm and selection decision mechanism.

The operation of DCCapsNet is as follows. In Conv 1, the input color image is first convolved with 256 convolution kernels of 3 × 3 size, and the convolution step is 1. The ReLu activation function is also used during the coiling operation. In Conv 2, dilated Inception module is used to carry out multi-scale convolution operation for the preliminary features obtained by Conv 1 convolution, and the convolution step is 1, so as to obtain the output results of the Conv 2 layer. In Conv 3, further carry out a dilated Inception module on the features obtained by Conv 2 convolution layers. In the primary capsule layer, vectorize the output results of Conv 3 layer. Ten groups of different convolutional kernels are adopted, and each group of coil-product kernels contained different convolutional kernels. The step of convolution is set as 1, and the activation function of this convolution operation is ReLu. After this step, the low-level feature is obtained, which is a vector of 1 × 10.

Dilated Inception module is composed of 1 × 1, 3 × 3, 5 × 5, and 7 × 7 convolutional kernels and a 3 × 3 maximum pooling in parallel. Its advantage is that four receptive fields with four sizes are used to extract the multi-scale features without increasing the parameters of the kernels individually at each stage of the network. Multi-scale kernels have better feature expression effects on the input complex images, so dilated Inception module has a better feature expression ability by the parallel configuration of the kernels. To test DCCapsNet on disease leaf images, the *k*-dimension feature vectors extracted by the capsule subnetwork are input into the Softmax classifier, which is described as follows:


(7) $$P(Y=i|x)=Soft\max(Y_{i})=\frac{exp(\varpi_{i}Y_{i})}{\sum_{i=1}^{K}exp(\varpi_{k}Y_{k})}$$

where *P* is the probability that the feature vector *x* belongs to the *i*th category, *K* is the total number of categories, *ϖ* is the weight items, and *y*_*i*_ is the corresponding label of the *i*th training sample.

The average recognition rate of apple disease experiments is often adopted to test the network performance. The test images in each class are used to measure the classification accuracy, which is calculated as follows:


(8) $$Accuracy=\frac{\text{Number of disease leaf images correctly identified}}{\text{Total number of test disease leaf images}}$$

The number of floating point operations (FLOPs), including multiplication and addition, depends on the model and can be used to evaluate model complexity. It is used as a criterion to assess the complexity of the model. To compute the number of FLOPs, suppose the convolution is implemented as a sliding window and the nonlinearity function is computed for free. For convolution layers, the FLOPs are computed as


(9) $$FLOPs\text{=(}2C_{in}K^{2}-1)HWC_{out}$$

where *H*, *W*, and *C*_*in*_ are the height, width, and the number of channels of the input feature map, respectively; *K* is the kernel size (assumed to be symmetric); *C*_*out*_ is the number of output channels.

For fully connected layers, the FLOPs are computed as follows:


(10) $$FLOPs\text{=(}2S_{in}-1)S_{out}$$

where *S*_*in*_ is the input dimensionality or the number of input neurons and *S*_*out*_ is the output dimensionality or the number of output neurons.

The FLOPs of the model are the sum of the FLOPs of the convolution layers and fully connected layers.

## Experiments and analysis

In this section, many experiments of apple disease recognition are conducted to validate the proposed method DCCapsNet and compared with improved convolutional neural network (ICNN) (Yan et al., 2020), VGG-ICNN (Thakur et al., 2022), LAD-Net (Zhu et al., 2022), and RegNet (Li et al., 2022). The comparative experiments and results are analyzed and discussed. The experimental configuration is shown in **Table 1**.

**Table 1:** Experiment configuration.

Experimental configuration	Parameter value
Processor	Intel Xeon E5-2643v3@3.40GHz
Graphics card	GTX2080Ti11 GB 64 GB
Memory	32 GB
Disk	100 GB
Deep learning framework	PaddlePaddle 1.8.4
Operating system	Ubuntu 16.04.1 LTS (64 bit)
Other tools	Python 3.7.1 CUDA Toolkit10.0 Pytorch

### Dataset

**Figure 5:**
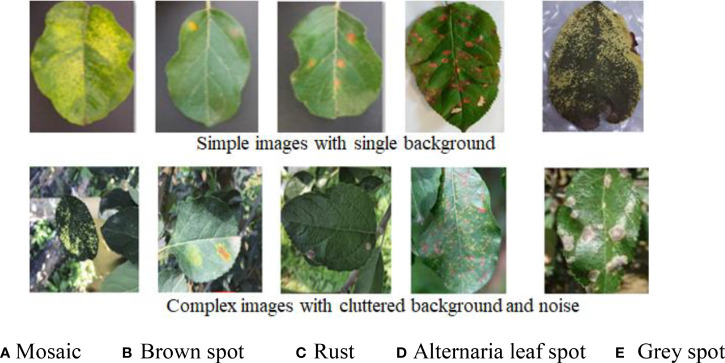
Ten image examples of five kinds of diseases. **(A)** Mosaic. **(B)** Brown spot. **(C)** Rust. **(D)** *Alternaria* leaf spot. **(E)** Gray spot.

The dataset of apple disease leaf images built by Northwest A&F University was used in the experiment. The dataset contains 26,377 images of five common apple disease leaves taken by BM-500GE color camera in an outdoor environment and laboratory environment. The data distribution are shown in **Table 2**. The dataset is randomly divided into a training set and a test set, in which the training set is used for training parameters, and the test set is used to verify the model. Five simple disease leaf images and five complex disease leaf images are shown in **Figure 5**.

**Table 2:** Apple disease leaf image distribution.

Apple leaf disease	Dataset	Training set	Test set
Mosaic	4,875	3,412	1,463
Brown spot	5,655	3,958	1,697
Rust	5,694	3,985	1,709
Gray spot	4,810	3,367	1,443
Spotted leaf litter	5,343	3,740	1,603
Total	26,377	18,462	7,915

**Figure 6:**
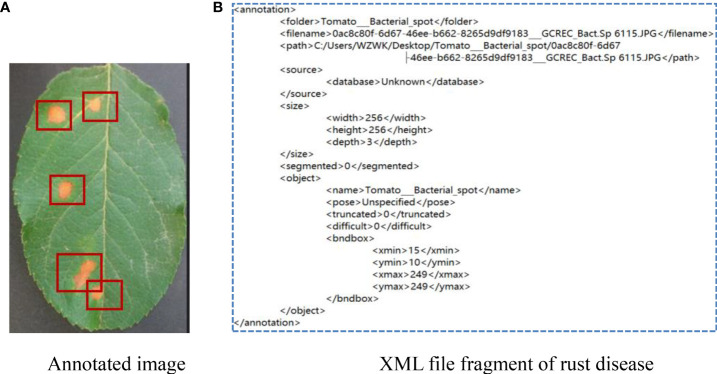
Annotation of apple rust disease leaf image. **(A)** Annotated image. **(B)** XML file fragment of rust disease.

As can be seen from **Figure 5**, the color and texture of rust and brown spots are similar with little difference. Due to different shooting conditions and complex backgrounds, the same subclasses may be affected by a single leaf or a cluster of leaves, leading to a large gap within classes. Therefore, a CNN-based method has a high probability of misjudgment in the process of disease identification. Image annotation is a crucial step in building the dataset. It is used to mark out the location and category of diseased spots in infected leaves. In this section, a tool has been developed to annotate images through rectangular bounding boxes. With the use of the annotation tool and the knowledge of experienced agriculture experts, areas of diseased spots in the image can be accurately labeled. When the annotation is complete, an XML file is generated for each image, which includes the types of diseased spots and their locations. The annotated image is shown in **Figure 6A**, and the infected areas are surrounded by boxes. **Figure 6B** is a fragment of the generated XML file, in which the disease name of rust is described and the location of diseased spots is determined by the upper left and lower right coordinates of the box.

### Experimental results

Experimental parameters are set as follows. Batch size is 16, the number of iterations is 3,000, the initial learning rate is 0.0005, and the momentum is 0.9. As the number of iterations increases, the learning rate is decreased by 0.05 times. If the loss of the network does not decrease after 10 iterations during training, stop the training. Each image is uniformly normalized to 512 × 512. The network parameters are initialized to generate weight parameters with a mean value of 0 and variance of 1, conforming to normal distribution. The average recognition accuracy is used to measure the performance of the network.

**Figure 7:**
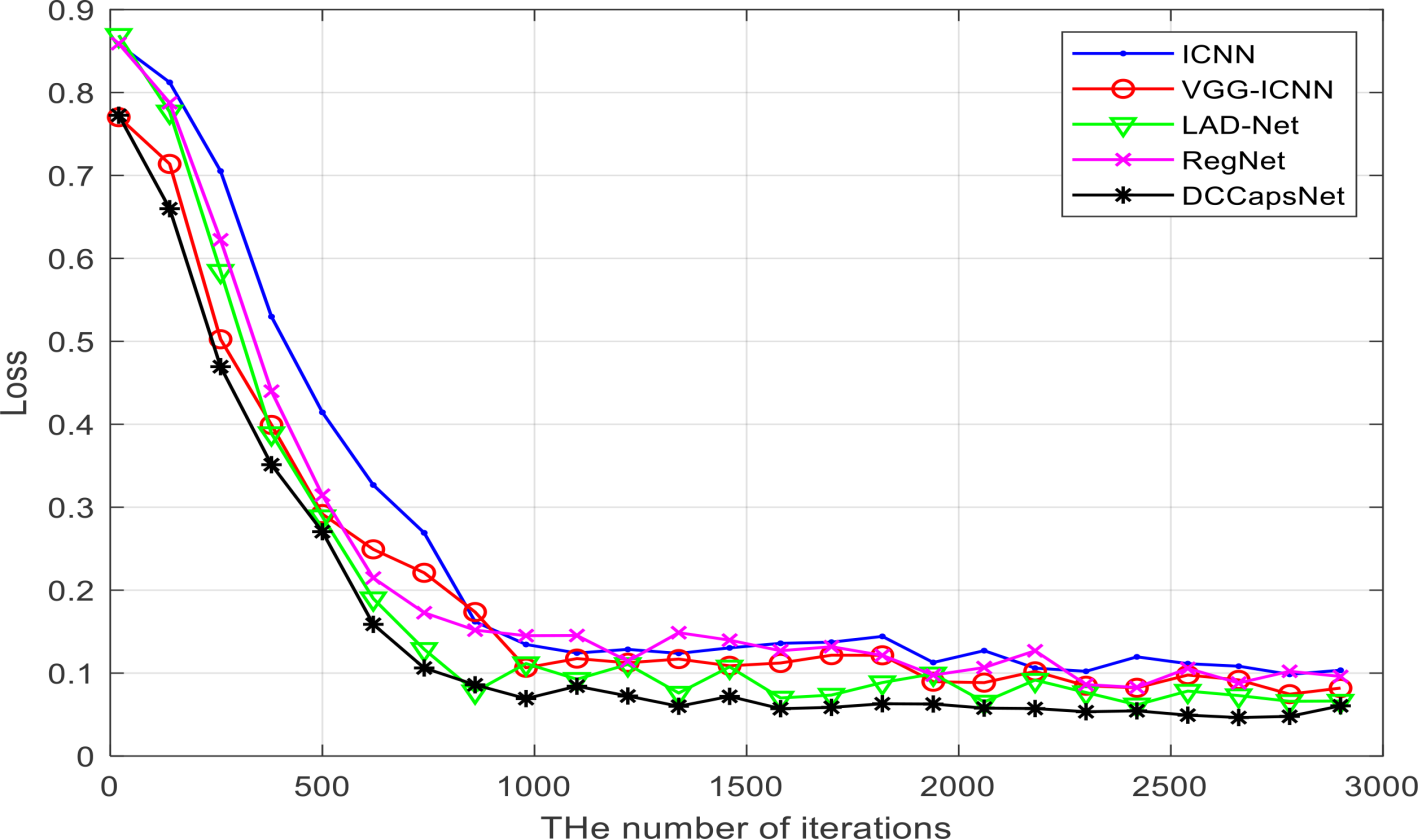
Losses of five networks versus training iterations.

DCCapsNet and four comparative deep learning models—ICNN, VGG-ICNN, LAD-Net, and RegNet—are trained on the image training set of apple disease leaves, from the beginning of the model training to convergence, so as to ensure that the training conditions of these models are the same. Each model is trained from the beginning until the model converged, and the training conditions of each model are guaranteed to be the same for a fair comparison. Their training losses versus the number of training iterations on the training set are shown in **Figure 7**, which can more intuitively display the performance changes of these models in the training process.

It can be seen from **Figure 7** that DCCapsNet has better convergence performance and recognition performance than other networks, and its convergence is relatively fast; the change in trend after 1,000 training iterations is relatively stable. Within the 3000th training iteration, all models converge basically, and before the 1000th training iteration, the loss of each network model decreases greatly, and the loss of each network model shows a downward trend as a whole. After 2,000 training iterations, the convergence performances of all models are improved and tend to be stable.

The apple disease recognition experiments are carried out with a fivefold cross-validation scheme. To be fair, four trained models are chosen after 3,000 training iterations to identify the leaf disease images in the test set. Their recognition results are shown in **Table 3**.

**Table 3:** The recognition results of ICNN, VGG-ICNN, LAD-Net, RegNet, and DCCapsNet.

Method	ICNN	VGG-ICNN	LAD-Net	RegNet	DCCapsNet
Pixel Seg. accuracy (PA)	89.12	91.11	92.17	89.64	93.16
FLOPs (G)	44.5	45.7	42.5	27.4	41.8
Training time (h)	7.51	6.41	7.17	6.50	3.44
Testing time (s)	3.18	2.82	3.19	3.73	2.51

From **Table 3**, it can be seen that DCCapsNet achieves the highest identification accuracy of 93.16%. Compared with ICNN, VGG-ICNN, LAD-Net, and RegNet, the recognition accuracy is improved by 4.04%, 2.05%, 0.99%, and 3.52%, respectively. DCCapsNet has fewer FLOPs and has higher PA than other models except for RegNet. RegNet is a lightweight convolutional network with 5.2M training parameters and has the least FLOPs because it aims to design spaces and find some network design principles, rather than just search for a set of parameters.

To verify the effectiveness of dilated Inception modules, several kinds of experiments are set up by introducing several Inceptions and dilated Inceptions into the convolution subnetwork of CapsNet. The modified networks are similar to DCCapsNet. The structures of Inception and dilated Inception are shown in **Figures 4A, B**. The experimental conditions are the same as above. The results of CapsNet and modified CapsNet are shown in **Table 4**.

**Table 4:** The results of CapsNet and modified CapsNet with different Inception modules.

Insert module into CapsNet	Accuracy	Training time
0 Inception, i.e., CapsNet	82.63	8.12 h
1 Inception	86.52	6.74 h
2 Inceptions	89.73	5.25 h
3 Inceptions	90.14	5.97 h
1 dilated Inception	90.15	4.76 h
2 dilated Inceptions, i.e., DCCapsNet	93.16	3.44 h
3 dilated Inceptions	93.18	4.61 h
1 Inception and 1 dilated Inception	92.06	5.11 h
2 dilated inceptions with 4 dilated rates	93.11	3.83 h

From **Table 4**, the conclusions obtained are summarized as follows. In general, adding convolutional modules can improve the recognition rate, while adding dilated Inceptions can further increase accuracy and reduce model training time. The main reason is that, compared with Inception, dilated Inception has four different-scale convolutional kernels without increasing additional training parameters, which can extract multi-scale features by applying different convolutional kernels in parallel and cascading their output feature maps. Its advantage is that there is no need to set the parameters of the convolutional kernels separately in each stage of the network. Multi-scale convolution has a better feature expression effect on the irregular disease leaf image, so Inception can have better feature expression ability through the parallel configuration of the convolution kernel. Dilated Inception is superior to Inception because it has different convolutional kernels with different respective fields without increasing training parameters.

From **Table 4**, it is also seen that the accuracy rates show an upward trend versus adding Inception or dilated Inception modules, and dilated Inception is better than Inception. However, adding three dilated Inception modules can greatly improve the identification accuracy while increasing the long training time. However, the addition of three dilated Inception modules can slightly improve the accuracy of recognition while greatly increasing the training time. Dilated Inceptions with four dilated rates have five different convolution kernels, such as 1 × 1, 3 × 3, 5 × 5, 7 × 7, and 9 × 9. When two dilated Inceptions with four dilated rates are added, the accuracy decreases instead of improving, indicating the dilated Inception module with convolution kernel 9 × 9 is not suitable for the image classification of disease leaves. Finally, the dilated Inception with dilated rate *r* = 1, 2, and 3 is selected.

**Figure 8:**
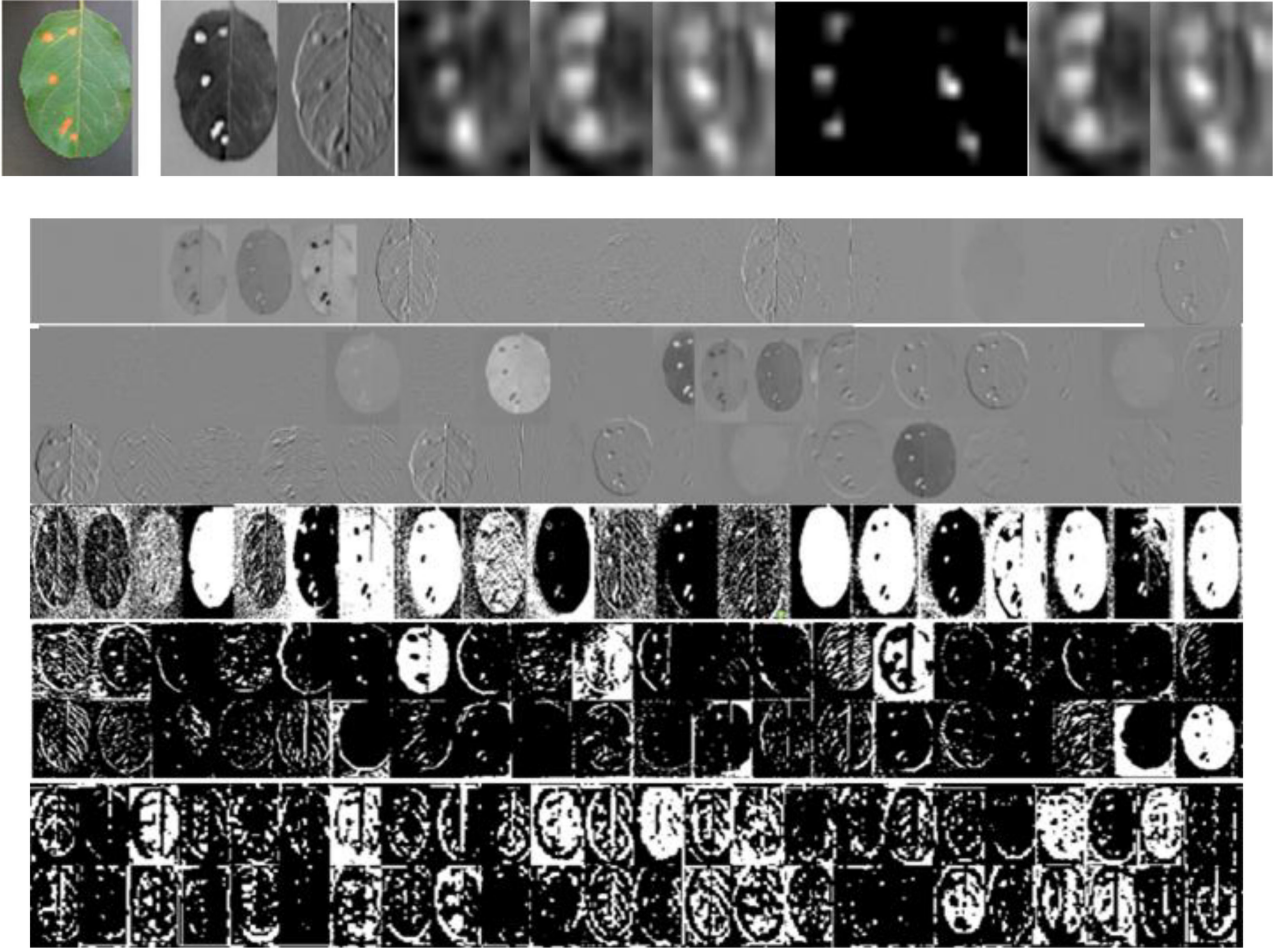
An original image and its feature map examples in different convolutional layers.

To verify the effect of the dilated Inception module on multi-scale features, **Figure 8** shows the visualization of convolutional feature maps of DCCapsNet. From **Figure 8**, it can be seen that DCCapsNet can obtain the multi-scale and multi-level feature by dilated Inception with three dilated rates.

### Result analysis

The results of **Figure 7** and **Tables 3**, **4** show that DCCapsNet has the highest recognition rate and the least FLOPs except for RegNet. The reason is that it makes use of the advantages of dilated Inception module and CapsNet. RegNet has the fewest FLOPs, but its recognition rate is lower but slightly higher than that of ICNN. LAD-Net is the next best because it uses LAD-Inception and attention mechanism to enhance the ability to extract multi-scale features of different sizes of disease spots and replaces a full connection with global average pooling to further reduce parameters. Although it is a lightweight model, it has little higher FLOPs than DCCapsNet due to the attention mechanism. VGG-ICNN is better than ICNN because it has few training parameters and has three Inception v7 blocks to extract the multi-scale features.

The result validates that when the depth of the network reaches a certain level, increasing convolutional layers of the network again is not as significant as expected, but as the depth of the network model increases, the model becomes more complex and the training time becomes longer. Therefore, ICNN is not easy to converge. Compared with ICNN and RegNet, DCCapsNet has better convergence performances due to the multi-branch parallel structure of dilated Inception, indicating that a multi-branch network is superior to a single-branch network in the disease identification task. It can extract multi-scale image features. Compared to VGG-ICNN and LAD-Net, DCCapsNet adds two dilated Inception modules that can extract rich features and overcome well the adverse effects of complex background environments and disease spots.

## Conclusion

CNN focuses on detecting important features of the input image and obtains invariance by pooling but loses some local information. Its output is only one scalar value, while the output of CapsNet is a vector, which not only can represent the characteristics of the input image but also can include the direction and state of the target. It is suitable for irregular disease leaf image classification, but its recognition accuracy is not high because there is only one convolutional layer. To improve accuracy, a DCCapsNet is constructed for apple leaf disease identification. Multi-scale classification features are extracted by adding two dilated Inception modules into CapsNet. The results on the apple disease leaf image dataset show that DCCapsNet is superior to other networks in recognition rate and training performance. This method has stronger practical application capabilities to promote the development of intelligent management systems for crop diseases in the field. In the future, we will embed this work into a smartphone-based disease diagnostic system for farmers in remote places.

## Data availability statement

The raw data supporting the conclusions of this article will be made available by the authors, without undue reservation.

## Author contributions

CX designed and performed the experiment, analyzed the data, trained the algorithms, and wrote the manuscript. CX and XW collected data. SZ selected the algorithm and monitored the data analysis. XW and SZ conceived the study and participated in its design. All authors contributed to this article and approved the submitted version.

## Funding

This work is supported by the National Natural Science Foundation of China (Nos. 62172338 and 62072378).

## Conflict of interest

The authors declare that the research was conducted in the absence of any commercial or financial relationships that could be construed as a potential conflict of interest.

## Publisher’s note

All claims expressed in this article are solely those of the authors and do not necessarily represent those of their affiliated organizations, or those of the publisher, the editors and the reviewers. Any product that may be evaluated in this article, or claim that may be made by its manufacturer, is not guaranteed or endorsed by the publisher.
